# Histological changes of immediate skin expansion of the distal limb of rats

**DOI:** 10.14202/vetworld.2018.1706-1711

**Published:** 2018-12-20

**Authors:** Ahmed Khalaf Ali, Adamu Abdul Abubakar, Ubedullah Kaka, Zamri Radzi, Nurul Hayah Khairuddin, Md Sabri Mohd Yusoff, Mohamad Yusof Loqman

**Affiliations:** 1 Department of Companion Animal Medicine and Surgery, Faculty of Veterinary Medicine, Universiti Putra Malaysia, 43400 Serdang, Selangor, Malaysia; 2 Department of Surgery and Theriogenology, College of Veterinary Medicine, University of Mosul, Mosul, Iraq; 3 Institute of Tropical Agriculture and Food Security, Universiti Putra Malaysia, 43400 Serdang, Selangor, Malaysia; 4 Department of Paediatric Dentistry and Orthodontics, Faculty of Dentistry, Universiti Malaya, 50603 Kuala Lumpur, Malaysia; 5 Department of Farm and Exotic Animals Medicine and Surgery, Faculty of Veterinary Medicine, Universiti Putra Malaysia, 43400 Serdang, Selangor, Malaysia; 6 Department of Veterinary Pathology and Microbiology, Faculty of Veterinary Medicine, Universiti Putra Malaysia, 43400 Serdang, Selangor, Malaysia

**Keywords:** histology, rats, skin expansion, tissue expander

## Abstract

**Aim:**

Tissue expansion is an applicable technique to reconstruct many surgical defects. The aim of this research was to evaluate the histological changes caused by immediate skin tissue expansion in rats as an animal model.

**Materials and Methods:**

Immediate skin tissue expansion in 18 adult female rats was performed using three different sizes (small, medium, and big) of polymethylmethacrylate tissue expanders at the dorsal surface of the metatarsal area of the right limb. The contralateral limb was served as the control. The tissue expanders were surgically implanted and kept for 15 days.

**Results:**

The immediate skin expansion resulted in histological changes such as the increased thickness of the epidermal layer, the reduction of the dermal layer, an elevated number of fibroblast as well as increased vascularity. Furthermore, skin adnexal structures such as hair follicles and sebaceous glands were farther apart.

**Conclusion:**

The rat skin was able to rapidly adjust and compensate against a specific range of immediate mechanical expansion. The histological changes suggest that the tissues were prepared to withstand the increased external forces, in addition to create possibly additional skin in a relatively short-term period.

## Introduction

Tissue expansion is an applicable technique which is used for the reconstruction of many surgical defects with additional local tissue attained by expansion process when primary closure is not feasible [1]. Expansion technique creates extra expanded tissue which ideally matches the color, texture, and hair bearance of the nearby skin defect [2].

Many histological changes of the epidermal layer of the skin are encountered as a result of tissue expansion such as increased epidermal thickness, hyperkeratosis, acanthosis, and increased mitotic activity. Electron microscopic examination reveals a more undulated basal lamina. The bundles of tonofilaments of tonofibrils in the basal and prickle (Malpighian) cell layers of the expanded skin are larger than those of the normal skin. Furthermore, reduced intercellular spaces in all layers of the epidermis of the expanded skin, as well as undulation of the basal lamina are noticed which may be attributed to the increased epidermal mitotic activity [3]. In relation to the skins’ dermal layer, significant dermal changes occur in response to tissue expansion such as a marked decrease in the thickness of the dermis, presence of large bundles of thin compacted collagen fibers with normal cross-banding and periodicity in the papillary and reticular dermis, and increased number of fibroblasts. In addition, the presence of myofibroblasts after several weeks of expansion suggests the contractile function of the dermis in response to the progressive expansion process. There are also clusters of thick and compact elastic fibers observed. The adnexal structures, such as sweat glands and hair follicles, are separated without quantitative and functional change during tissue expansion. Fibers are either multiple or single, and the majority are straight, and some have coiled or bent appearance. Other changes involve inflammatory reactions and infiltration of the foreign-body giant cells within the capsule, epidermis, or dermis. Rapid angiogenesis by the increased number of vessels located at the junction between the capsule and host tissue also can be observed [4]. The microscopic changes caused by tissue expansion process such as increased epidermis thickness, decreased dermis thickness, thinning hypoderma, elastic fiber rupture, tiny thrombus formation, fibrinolysis, and decreased viscoelasticity of skin are recessive changes [5].

Tissue expansion is a feasible alternative technique to reconstruct relatively big skin deficits due to diseases or injuries. However, there is still limited information with regard to the application of the immediate skin expansion technique in small animals. This study aimed to evaluate the microscopic changes of skin layers with regard to the skin thickness and cellular structure numbers caused by immediate tissue expansion in rats as an animal model.

## Materials and Methods

### Ethical approval

All procedures performed were approved by the Institute Animal Care and Use Committee (IACUC) of University Putra Malaysia (Project approval number: UPM/IACUC/AUP-R018/2015 and UPM/IACUC/AUP-R063/2015).

### Bone cement tissue expander polymethylmethacrylate (PMMA)

Three different sizes of rectangle tissue expander of constant length and width were used to induce immediate tissue expansion in rats (5 mm × 3.50 mm × 3.50 mm, 5 mm × 3.50 mm × 4.00 mm, and 5 mm × 3.5 mm × 4.50 mm). The expanders were made of bone cement (PMMA), which is an acrylic polymer prepared by mixing two components, namely a liquid monomer and a powdered polymer (Orthocryl, Dentaurum, GmbH and Co. KG, Germany). Polymerization of the liquid monomer around the pre-polymerized powder particles resulted in hardened PMMA [6,7]. These expanders were sterilized by autoclaving for 15 min at 121°C under 15 lb per sq inch pressure without any physical or chemical damage or change.

### Animals and implantation technique

All the experimental designs in this study were approved by the Institute Animal Care and Use Committee of University Putra Malaysia. In this study, 18 healthy Sprague-Dawley female rats, aged 8 weeks with body weight ranged from 140 to 180 g, were used to study skin tissue expansion at the dorsal surface of the metatarsal region.

**Figure-1:**
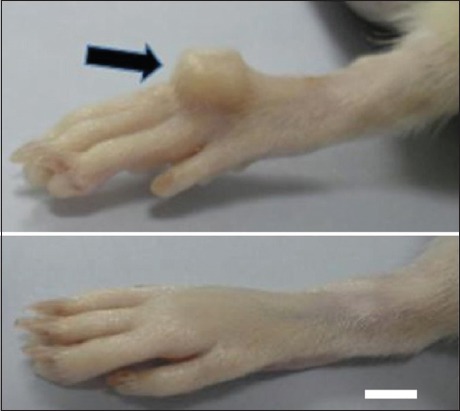
Skin tissue expansion of the right metatarsal region (black arrow) on the 14^th^ day following surgical implantation of polymethylmethacrylate expander in a rat. The normal contralateral leg (bottom) is shown as a comparison. Bar=0.3 cm.

Rats were divided into three groups. Each group consists of six animals. Small (3.50 mm height), medium (4.00 mm height), and big (4.50 mm height) PMMA expanders with constant length (5 mm) and width (3.5 mm) were used, respectively, in the different groups of animals. The expanders were inserted subcutaneously at the dorsal surface of the right metatarsal region and kept for 14 days. The control was the normal skin of the left metatarsal regions of each animal. Ketamine (Vetoquinol, U.K) and xylazine (Troy Laboratories Pty Ltd., Australia) were used for anesthesia at a dose of 90 mg/kg and 10 mg/kg of body weight, respectively. The surgical site was aseptically prepared using 40% chlorhexidine scrub and disinfected with 10% povidone-iodine. The rats were positioned in the left lateral recumbency. The surgical site was shaved and prepared for aseptic surgery. 1 cm vertical skin incision was made at the midpoint of the dorsal aspect of the right metatarsal area, and a subcutaneous pocket was created by blunt dissection of the skin tissue. The expander was implanted subcutaneously at the dorsal aspect of the metatarsal area using tunnel approach. The skin incision was sutured in simple interrupted pattern using 4/0 nylon (Ethicon, Johnson and Johnson, USA) suture material (Figure-1).

### Tissue processing for histological evaluation

Skin specimens were fixed using 10% buffered formaldehyde solution. The specimens were embedded in paraffin, stained with hematoxylin-eosin, and examined under a light microscope. A computer-aided digitalizing pad and software (Panoramic Viewer) (3DHISTECH Ltd., Hungary) were used to estimate both epidermal and dermal thicknesses by averaging 10 measurements for each layer at 4× magnification. The distance from the dermoepidermal junction to the base of the reticular dermis was considered in the measurement of the dermal thickness. The hair follicles and sebaceous glands number were counted in randomly selected four fields of each slide at 10× magnification. The count number of the fibroblast of randomly selected four fields of each slide of both expanded and normal skin samples at 40× magnification was done.

### Statistical analysis

The results were analyzed statistically using Student’s t-test and analysis of variance methods to identify the histological differences between expanded and normal skin samples. The data were considered statistically significant at p<0.05.

## Results

**Figure-2:**
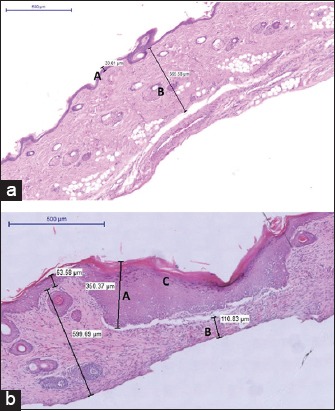
Photomicrograph of normal skin tissue of the metatarsal area of a rat (top) and of the same area that underwent immediate skin expansion (using medium size polymethylmethacrylate expander) (below) shows normal thickness of the epidermis (a) and dermis layer (b) in the normal skin and the expanded skin. C-hyperkeratosis (H E stain 200×).

**Figure-3:**
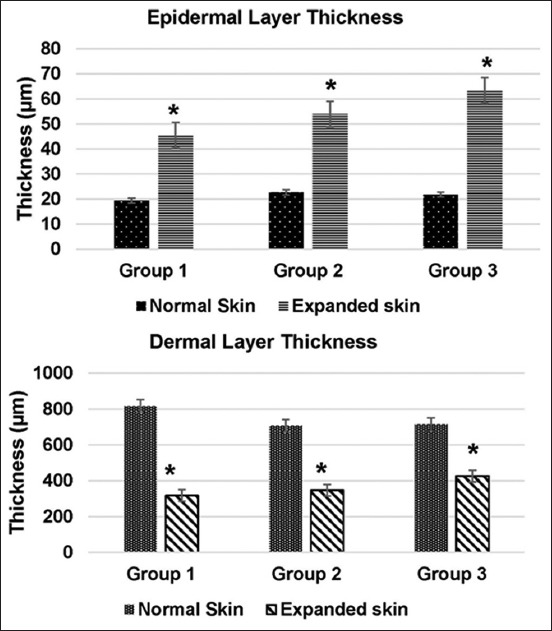
The difference in the thickness of the epidermis layer (top) and dermal layer (bottom) of the immediate expanded skin tissue of the metatarsal area of Group 1 (small size), Group 2 (medium size), and Group 3 (big size polymethylmethacrylate expanders) in rats. *Denotes significant difference of epidermis layer thickness of the expanded tissue compared with the normal skin tissue within groups at p<0.05. There was no significant difference among the expanded skin of the different groups at p<0.05.

**Figure-4:**
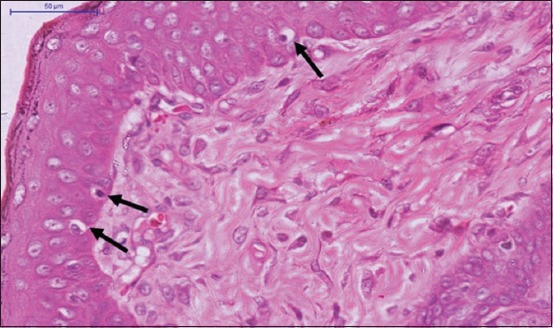
Photomicrograph of immediate expanded skin tissue (medium polymethylmethacrylate expander) of the metatarsal area of a rat shows the increased melanocytic activity (black arrows) (HE stain 400×).

Immediate skin expansion resulted in the significant increase (p<0.05) in thickness of the epidermal layer and decrease (p<0.05) in thickness of the dermal layer in comparison with the normal skin. Increased mitotic activity was expressed by hyperkeratosis (Figures-2 and 3). Increased melanocytic activity was also noticed in the samples of the expanded skin (Figure-4).

The epidermal thickness (µm) of normal skin samples (mean±SE [standard error]) of the Groups 1, 2, and 3 of rats was 19.4±2.39, 22.67±1.18, and 21.76±1.48, respectively, compared to 45.45±1.96, 53.87±19.00, and 63.35±14.40, respectively, of the immediately expanded skin samples. For all groups, the increased epidermal thickness of the expanded skin specimens was statistically significant (p<0.05) from their corresponding normal (control) skin specimens (Figure-3).

The dermal thickness (µm) of normal skin samples (mean ± SE) of the Groups 1, 2, and 3 of rats was 817.49±117.63, 707.24±26.93, and 714.31±64.93, respectively, and 317.88±57.92, 346.53±21.42, and 425.85±30.23, respectively, of the immediately expanded skin samples. For all groups, the decreased epidermal thickness of the expanded skin specimens was statistically significant (p<0.05) compared with corresponding normal (control) skin specimens (Figure-3).

**Figure-5:**
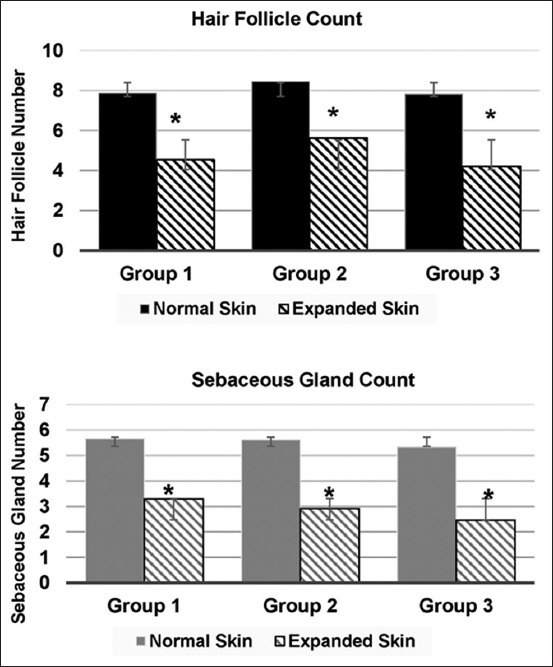
The difference in the number of the hair follicle (top) and sebaceous gland (bottom) of the immediate expanded skin tissue of the metatarsal area of Group 1 (small size), Group 2 (medium size), and Group 3 (big size polymethylmethacrylate expanders) in rats. *Denotes significant difference of the sebaceous gland number of the expanded tissue from the normal skin tissue within groups at p<0.05. There was no significant difference among expanded skin of different groups at p<0.05.

The decreased dermal thickness was compensated by the thickness of the fibrous capsule formed around the implant. Adnexal structures such as hair follicles and sebaceous glands became separated in the expanded skin, but they maintained their normal morphology. For all groups, the decreased number of the adnexal structures of the expanded skin specimens was statistically significant (p<0.05) compared with corresponding normal (control) skin specimens (Figure-5).

**Figure-6:**
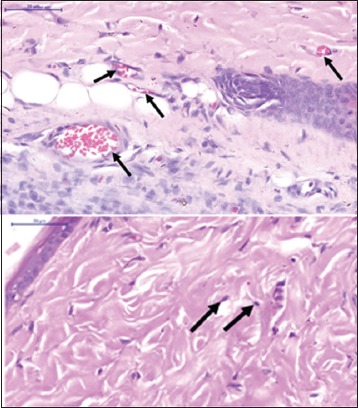
Photomicrograph of immediate expanded skin tissue (small polymethylmethacrylate expander) of the metatarsal area of a rat shows the increase in the number of blood vessels (black arrows) (HE stain 400×).

**Figure-7:**
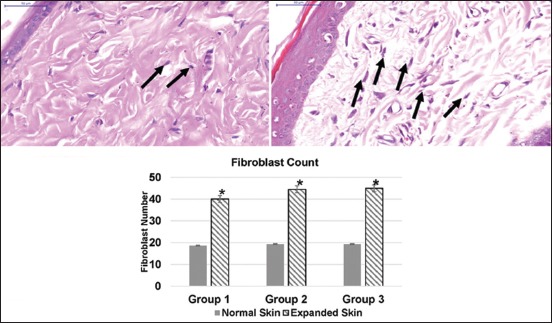
Photomicrograph of normal skin tissue (top) and immediate expanded skin tissue (medium polymethylmethacrylate [PMMA] expander) (below) of the metatarsal area of a rat shows the increased number of fibroblast (fibroplasia) (black arrows) in the expanded skin in comparison to the normal skin (HE stain 400×). The graph shows the difference in the number of the fibroblast cell of the immediate skin tissue expansion using of relatively small (Group 1), medium (Group 2), and big size PMMA expander (Group 3). *Denotes significant difference of the fibroblast number of the expanded tissue from the normal skin tissue within groups at p<0.05. There was no significant difference among the expanded skin of different groups at p<0.05.

There was an increased blood supply in the expanded skin to that of normal skin. It was shown by the clearly visible increase in size and number of vessels in the expanded skin as well as the vascularity of the capsule surrounding the implant (Figure-6). The increased number of fibroblast (fibroplasia) and increased collagen deposition in the dermis were the major tissue response to expansion process in comparison with the normal skin tissue (Figure-7). Large bundles of compacted collagen were observed in the expanded dermis. For all groups, the increased number of fibroblast of the expanded skin specimens was statistically significant (p<0.05) compared to corresponding normal (control) skin specimens (Figure-7).

## Discussion

Our study demonstrated an increased thickness of the epidermal layer and the decrease of the dermal layer thickness in response to immediate tissue expansion. The significant decrease of dermal thickness could be directly implicated to the immediate skin tissue expansion [8]. On the other hand, the epidermal hyperkeratosis and acanthosis represented the increase in mitotic activity in the expanded skin. The epidermal mitotic activity is regulated by the activation of growth factor pathways which is triggered by epidermal tension [9]. This mitotic activity is also regulated by cell density of the basal layer of the skin which is decreased by tissue expansion where greater cell proliferation is induced by lower cell density, leading to the growth of additional skin [10]. In spite of some tension transfer to the epidermis, the significant thinning of the skin can be attributed to the maximum tension to which the dermis is exposed during expansion, leading to a significant thickening of the epidermis and thinning of both dermis and subcutaneous tissue [11]. This result showed that the changes of epidermal and dermal layer thickness were one of the main microscopic features of immediate skin expansion in rats.

The results of the current study also showed an increased melanocytic activity in the expanded skin. This could be associated with the clinical hyperpigmentation [12], where hyperpigmentation was attributed to the hyperactivity of melanocytes during expansion and tends to be slowly reversible to normal in the event of expander or the mechanical stimulus removal. The immediate expansion of the skin also resulted in a significant decrease in the adnexal structures such as hair follicles and sebaceous glands, where they were found relatively farther apart. Inevitably, the immediate expansion of the skin could affect the cosmetic appearance of the skin to a certain extent due to the sparse distribution of the hair growth. As a result, immediate skin expansion could probably not produce the exact esthetic appearance of skin tissue as the normal skin.

The present result showed the increased number of fibroblast (fibroplasia) and collagen deposition in the dermis in comparison with the normal skin. Large bundles of compacted collagen were observed in the expanded dermis. Tissue expansion resulted in an increased number of fibroblasts and the presence of compacted collagen and angiogenesis as reported in another study [9]. The increase of collagen and extracellular matrix synthesized by the fibroblast is a response to either the pressure by the tissue expander or traction of the fibroblast cytoskeleton by the extracellular matrix [13]. The mechanical load stimulates the extracellular matrix components to switch cells between growth, differentiation, and involution, whereas the synthesis and secretion of intracellular products are evoked within 24 h after a single mechanical stimulus. Large bundles of compacted collagen observed in the expanded dermis represented abundant quantities of immature collagen produced by the elevated numbers of fibroblasts within the dermis. These histological changes appeared to be one of the major tissue responses to immediate expansion process and in this case for the creation of additional skin.

The increased vascularity of expanded skin was noticed in this study as compared to the normal skin. The realignment of vessels, the closing of arteriovenous shunts, neovascularization, and the depletion of neurohumoral vasoactive substances are the most factors seemed to be related to the explanation for increased vascularity in tissue expansion [4]. In this present study, the implantation of the expander (PMMA) could lead to the formation vascular fibrous capsule surrounding the implant as a result of tissue-foreign body reaction [1]. Therefore, the increased vascularity in immediate expanded skin is partly as the result of tissue reaction involving the increase in the skin vascularity by having a circulation exceeding that of the subdermal plexus.

The present study introduced a new method to induce immediate tissue expansion using different sizes of PMMA expanders. Other methods that were used previously in reported studies including the use of traditional tissue expander (e.g., silicon balloon) and hooks [4,12]. Besides that, the immediate skin expansion involving the distal rat’s limb region was performed and reported for the 1^st^ time in this present study. The skin thickness layers and number of skin structures and cells were quantitatively obtained and analyzed in comparison to descriptive observations of histological changes in the previously reported studies [4].

## Conclusion

The microscopic changes in rats’ immediate skin expansion showed that the tissue was able to rapidly adjust and compensate against a specific range of mechanical stretching. Increased epidermal thickness, reduced dermal thickness, an elevated number of fibroblasts, increased vascularity, and parallel orientation of collagen fibers were the main histological changes observed in response to tissue expansion. The histological changes suggest that the tissues were prepared to withstand the increased external forces, in addition to create possibly additional skin in the relatively short term although at the expense of plausibly the cosmetic outcomes such as the amount of hair growth per surface area.

## Authors’ Contributions

AKA conducted the study, drafted, and prepared the manuscript. AAA and UK assisted in the conduct of the study and manuscript preparation. ZR, NHK, and MSMY assisted in analyzing the results and the manuscript correction. MYL drafted the initial study plan and assisted in the data analysis, manuscript preparation, and correction. All authors read and approved the final manuscript.
